# Strategic aspects of higher education reform to cultivate specialists in diagnostic and biopharma industry as applicable to Predictive, Preventive and Personalized Medicine as the Medicine of the Future

**DOI:** 10.1186/s13167-015-0040-4

**Published:** 2015-09-15

**Authors:** М. Studneva, M. Mandrik, Sh. Song, E. Tretyak, I. Krasnyuk, Y. Yamada, A. Tukavin, A. Ansari, I. Kozlov, C. Reading, Y. Ma, K. Krapfenbauer, A. Svistunov, S. Suchkov

**Affiliations:** A.I.Evdokimov Moscow State University of Medicine and Dentistry, Moscow, Russia; I.M.Sechenov First Moscow State Medical University, Moscow, Russia; EPMA (European Association for Predictive, Preventive and Personalised Medicine), Brussels, Belgium; New York Academy of Sciences, New York, NY USA; ACS (American Chemical Society), Dallas, TX USA; AMEE (European Association for Medical Education), Dundee, UK; Department of Pharmaceutics, University of Florida College of Pharmacy, Gainesville, FL USA; Division of Ocular Diseases, Central Clinical Hospital No. 85, FMBA, Moscow, Russia; Department of Human Functional Genomics, Mie University Graduate School of Medicine, Mie, Japan; St. Petersburg State Chemical and Pharmaceutical Academy, Saint Petersburg, Russia; College of Science, King Saud University, Riyadh, Saudi Arabia; NeurMedix, San Diego, CA USA; College of Arts, Science and Business, Missouri University of Science and Technology, Rolla, MO USA; N.I.Pirogov Moscow Medical Research University, Moscow, Russia

**Keywords:** Predictive, Preventive and Personalized Medicine, Education, Companion diagnostics, Pharma industry, Innovation, Drug design, Targeting, Bioinformatics, Translational medicine, Integration of science and education

## Abstract

Predictive, Preventive and Personalized Medicine as the Medicine of the Future represents an innovative model for advanced healthcare and robust platform for relevant industrial branches for diagnostics and pharmaceutics. However, rapid market penetration of new medicines and technologies demands the implementation of reforms not only in the spheres of biopharmaceutical industries and healthcare, but also in education. Therefore, the problem of the fundamental, modern preparation of specialists in bioengineering and affiliated fields is becoming particularly urgent, and it requires significant revision of training programs of higher education practice into current medical universities. Modernization and integration of widely accepted medical and teaching standards require consolidation of both the natural sciences and medical sciences that may become the conceptual basis for a university medical education. The main goal of this training is not simply to achieve advanced training and expansion of technological skills, but to provide development of novel multifaceted approaches to build academic schools for future generations.

## Review

### Limitations of the current diagnostic and therapeutic paradigm

*Biopharma* as an industrial branch has always been considered as a strategic industry, being an important component of national security that includes the following typical enterprises and organizations:

- Chemical and pharmaceutical departments and research chemical and pharmaceutical institutes
- Supervising institutions and organizations for specific categories of medicinal products
- Departmental organizations (particularly of the Ministry of Defense) and so on
- Biopharmaceutical industries including production of active ingredients

Hence, a complete manufacturing chain has been available—from the selection of active biological molecules and potential pharmacotherapeutic targets to the production of the final product and marketing thereof. This requires corresponding personnel support and a professional preparation system, including higher educational institutions, specialized secondary schools, and post-graduate education (post-graduate qualification, doctoral degree, career development system) [1].

Development of medicinal products and preparations and diagnostic instruments of each next generation is a long, science-intensive and costly process. The junction of complex biosystems theory and translational medicine has led to the rampant development of *systems biology*, resulting in the active introduction of principally new approaches into the practice of specialists in pharmaceutical design: these are now oriented to the creation of market products with exclusively high-tech level.

The area of use of such products has been greatly expanded covering the segments of therapeutic-diagnostic (theranostic), predictive-prognostic, preventive-prophylactic, and rehabilitation instrumentation. This, in turn, has been dictated by the birth of technological platforms of principally new generations, on the one hand, and internal restructuring of the healthcare model replacing the corresponding segments with groundbreaking innovation technologies of Predictive, Preventive and Personalized Medicine (PPPM), on the other hand. And that is why today the existing market situation strongly requires not only the update of the biopharmaceutics engineering ideology and its technological inventory, but also the acceptance of more radical steps in terms of rotation of the personnel and renovation of the whole system infrastructure. Why so? The latter, having been capable of ensuring the bioengineering sectors of the pharmaceutical industry as an economy segment with personnel, is nowadays progressively and, more importantly, hopelessly and irreversibly aging, therefore requiring a radical reform from the pool of bioengineers [2].

The shortage of skilled personnel capable of solving principally new tasks is felt today even in the majority of developed countries including the regions with powerful biotechnological and biopharmaceutical clusters (USA, Scandinavia, Hong Kong, and others). And this happens in spite of the fact that over 50 % of the graduates of pharmaceutical departments of European universities are oriented to work in the specialized sectors of industries and affiliated structures thereof.

**Fig. 1:**
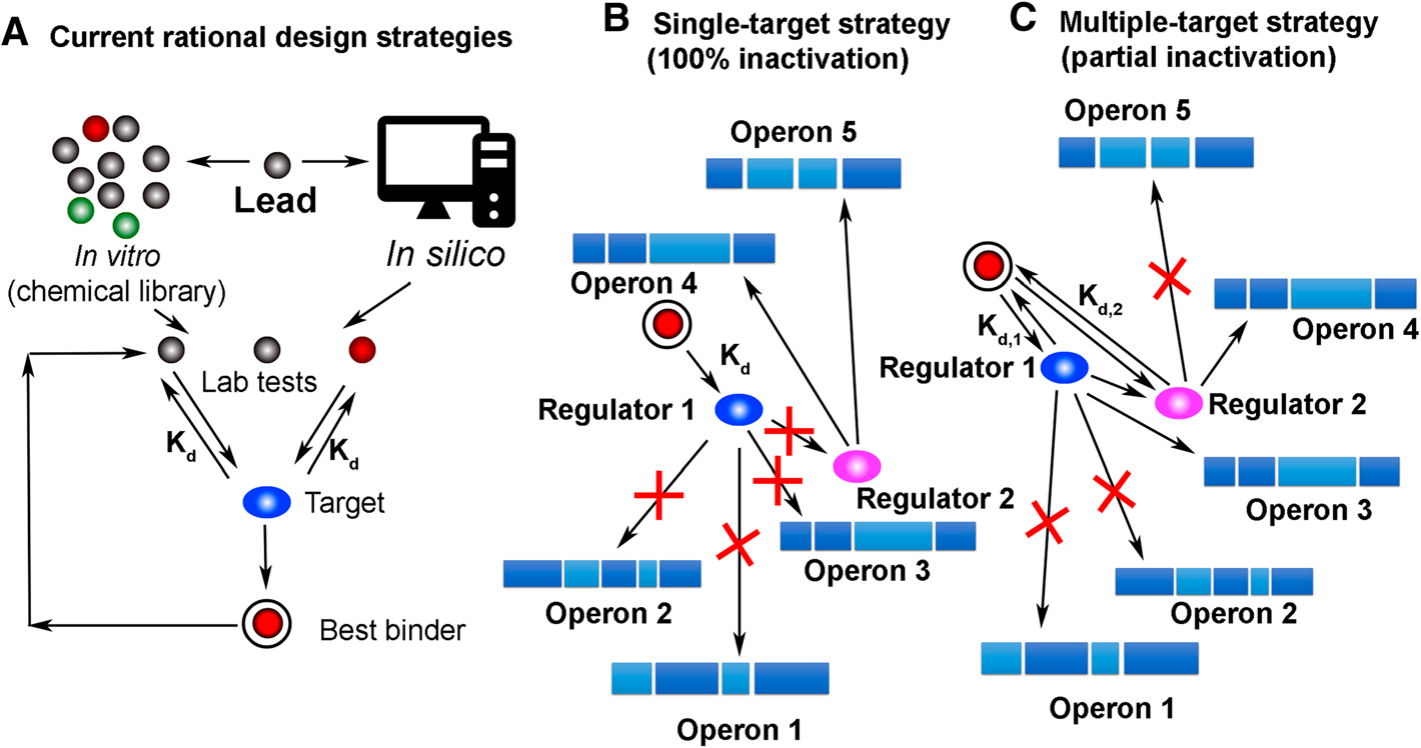
Multitargeting focused on realization of a therapeutic potential of the drug designed. **a** Use of high-performance screening and molecular modeling. A three-dimensional structure of the target explains a molecular mechanism of interaction of a ligand with protein; it is used for *molecular docking* or computer modeling of the ligand-protein interaction. The docking uses the three-dimensional structure as startup information (at this stage of technology development, it is usually conformationally fixed) and the ligand structure whose conformational flexibility and mutual arrangement with the receptor are modeled in the process of docking. The docking makes it possible to reduce the expenses and time due to conducting a procedure analogous to high-performance screening with the use of computer environment. This procedure is called *virtual screening* with the main advantage of which being that for real pharmacological tests, and it is not necessary to acquire a complete library consisting of millions of compounds, but only “virtual prototypes.” When the target is selected, the library of compounds may be used as a startup set of ligands. The task at this stage is to detect the compounds that after further modification and testing may offer a candidate compound for further testing on animals and people. **b** A pharmaceutical analogous to the ligand is bonded with a protein regulator but does not activate it. Complete inactivation of a pharmaceutical takes place. **c** A pharmaceutical simultaneously cooperates with several regulators, as a result of which its partial inactivation is observed. In the process, the protein regulators transmit information to the structural genes used to regulate protein synthesis

**Fig. 2:**
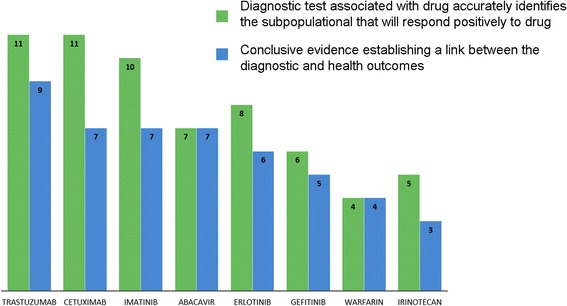
The role and place of the “companion” diagnostics as a special segment of the biopharmaceutical market. The diagram shows the ratio of the number of patients who have obtained a positive result upon receiving a pharmaceutical to the number of patients whose quality of life has drastically improved after termination of their therapy [20]

**Fig. 3:**
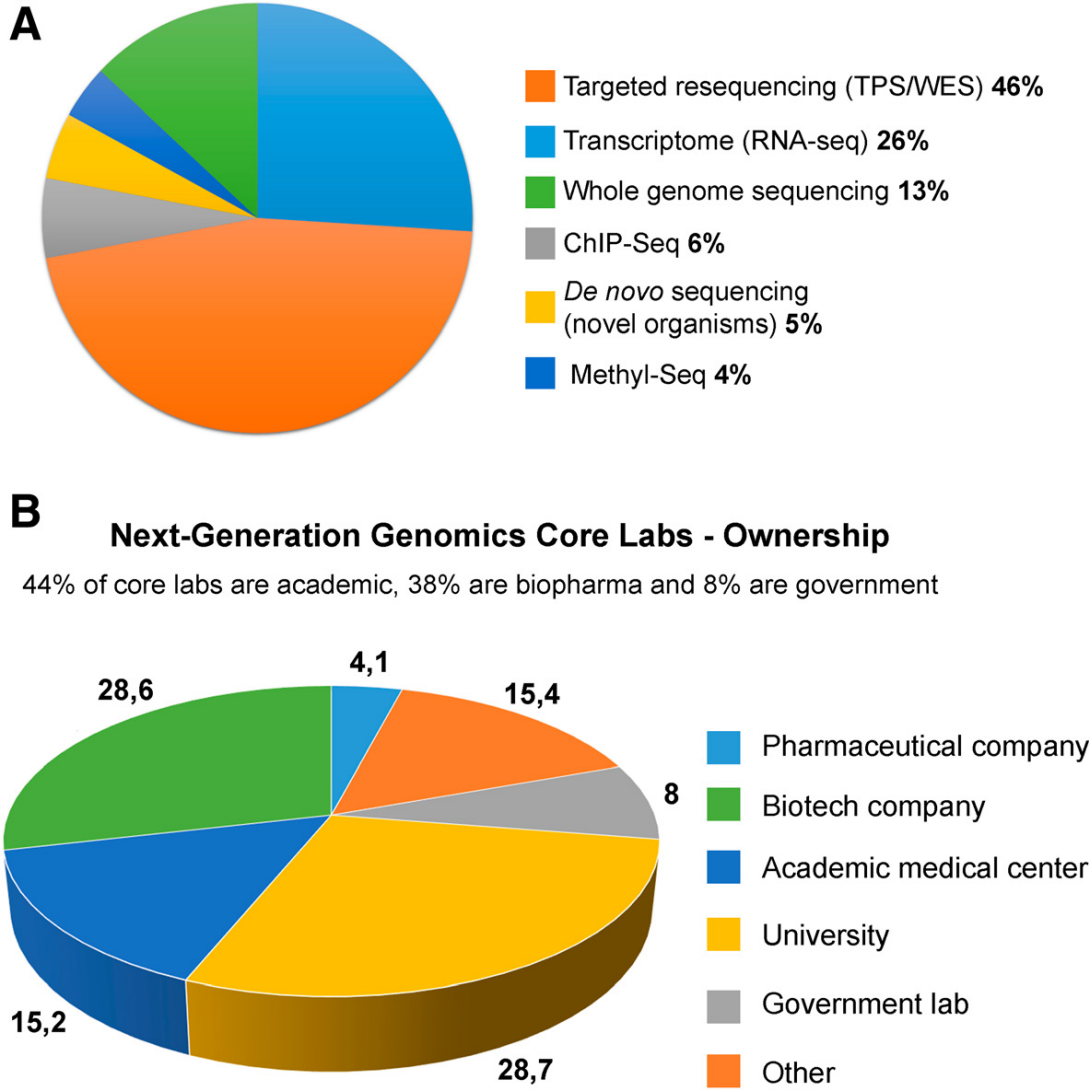
**a** Methods of next-generation sequencing (NGS). NGS relates to the determination of the initial sequence of DNA or RNA with further description of the primary sequencing item structure. This technology allows simultaneous reading of several genome sections which is the main difference as compared with the existing methods of sequencing. NGS is performed with the help of multiple chain extension cycles induced by polymerase or multiple oligonucleotide ligation. **b** The ratio of the number of new-generation laboratories dealing with of genomics. Note that 44 % of the laboratories belong to the higher education structures (in the education category), 38 % belong to the structure of biopharmaceutical enterprises, and 8 % of the laboratories belong to the government

**Fig. 4:**
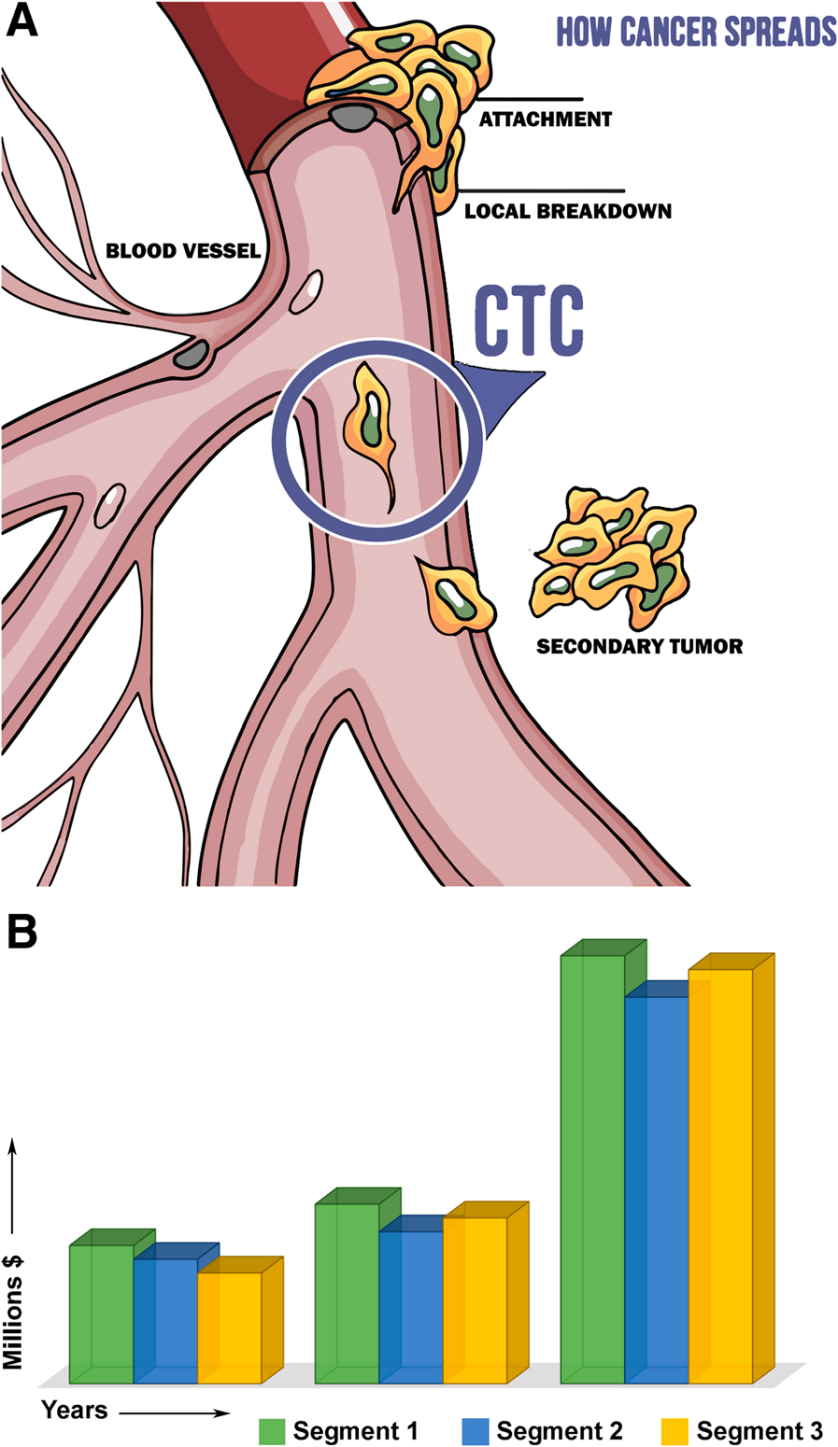
**a**, **b** Volumes of sales of CTC-products in the world market and prognosis for 2012–2018 in USD million. **а** The primary tumor cells are capable of breaking off from the main tumor mass and circulate with the blood flow in the organism; such cells are called circulating tumor cells (CTCs). A solution of diagnostic and predictive problems is provided by a special technology and device (CellSearch™) capable of detecting СТСs in the course of blood analysis. The principle of action of the device is based on the method of immunomagnetic separation of fractions, followed by fluorescent identification of CTCs. **b** Prognosis of СТС technology sales for the period of 2012–2018

Thus, with due account of increase in the labor market of biopharmaceutics and biopharmaceutical industries, the demand for qualified *research* and *production* personnel is essentially increasing. At the same time, the demand for sales managers and marketing specialists is evidently decreasing. The companies oriented for high-technology developments in creative segments, including (а) multitarget pharmacological structures oriented for the components being part of various signal paths (Fig. 1), (b) “companion” diagnostics (Fig. 2), (c) next-generation sequencing (NGS) (Fig. 3), or (d) screening for presence of circulating tumor cells (CTCs) in the blood (Fig. 4), and others are in need of creativity personnel to staff their search and R&D subdivisions.

Here, one talks about specialists of a new generation in principle, namely, specialists in the field of personalized genomics, proteomics, drug design, and bioinformatics.

Exactly these specialists are capable not only of engineering pharmacological structures and predictive-diagnostic instrumentation of new generations, but also of prognosticating selection of potential pharmacotherapeutic target candidates out of new (including earlier unknown) families by determining the production platforms and methods of quality assurance with them in the corresponding configurations of operation therewith [3].

The main terminology used in the pharmaceutical design consists of *pharmacotherapeutic target (PTT)* and *target pharmacological structure (TPS)* (drug).

**Fig. 5:**
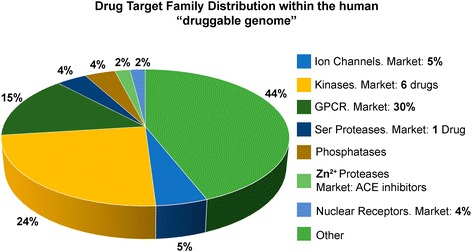
Families of potential pharmacotherapeutic targets in the human body. Kinases are enzymes acting as catalysts for the transfer of the phosphate group from the adenosine triphosphate (ATP) molecule to various substrates. Receptor tyrosine kinases (ТK) act as a catalyst for the transfer of the phosphate residue from ATP to tyrosine residue of specific cell protein targets. Non-receptor tyrosine kinases are located in the cell cytoplasm and are attached to cytoplasmic membrane on the inner side. An example may be the SRC protein which has one changed amino acid in it, inducing hyper-expression of genes and growth of the cell. An ion channel receptor is a pore-forming protein supporting the difference of potential that exists between the outer and inner sides of the cell membrane in live cells. The GPCR receptor is a G protein-coupled receptor (relates to the family of transmembrane proteins). Zinc proteases: zinc is a coenzyme of over 60 enzymes in human body. Nuclear receptors represent DNA-coupling transcriptional factors with conservative domain organization, the activity of which is controlled by lipophilic ligands, phosphorylation, and interaction with other proteins. Serine proteases are proteases used to modify other proteins by phosphorylation of serine residue. Phosphatases are enzymes used to dephosphorylate the substrate as a result of ester decomposition of phosphoric acid

*PTT* is a macromolecular biological structure supposedly associated with a definite function, the violation of which may cause formation of pathological shifts at the preclinical stage followed by clinical manifestation of a disease, which requires a specific action to prevent. The most frequent targets are receptors and enzymes (Fig. 5).

**Fig. 6:**
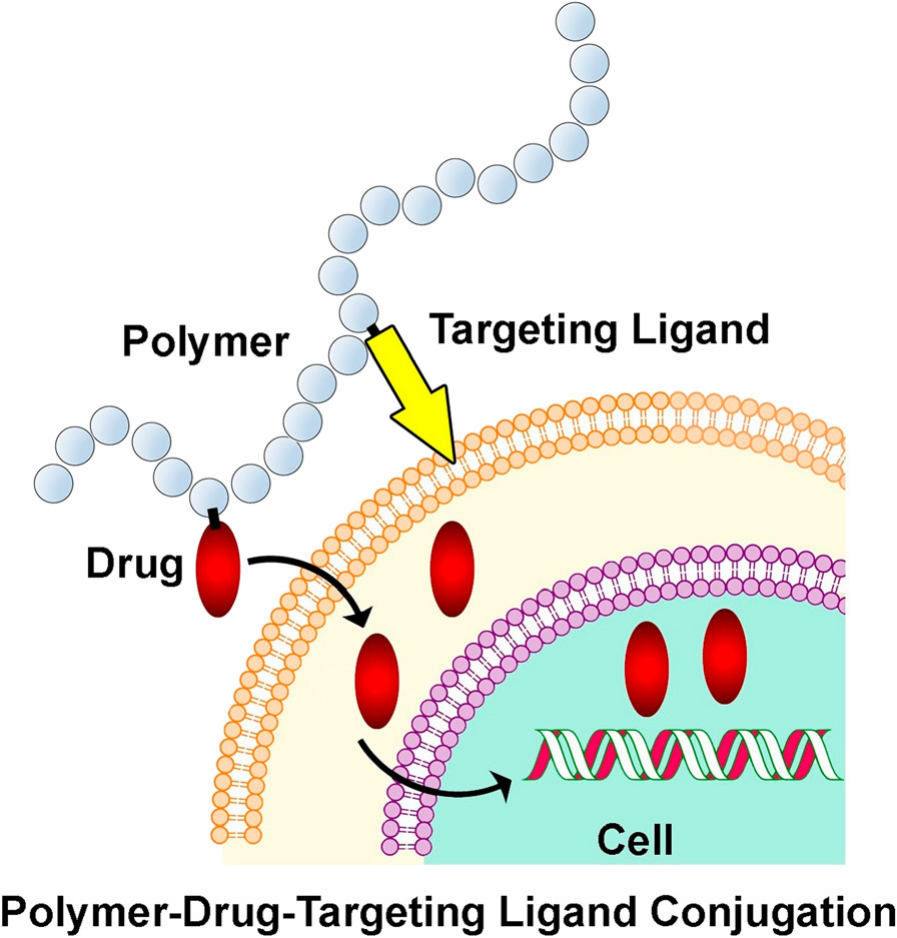
Interaction pattern in a polymer-pharmaceutical-ligand conjugation

*TPS* is a chemical compound (or biological product) specifically interacting with the target and capable of modifying the final cell response (Fig. 6).

**Fig. 7:**
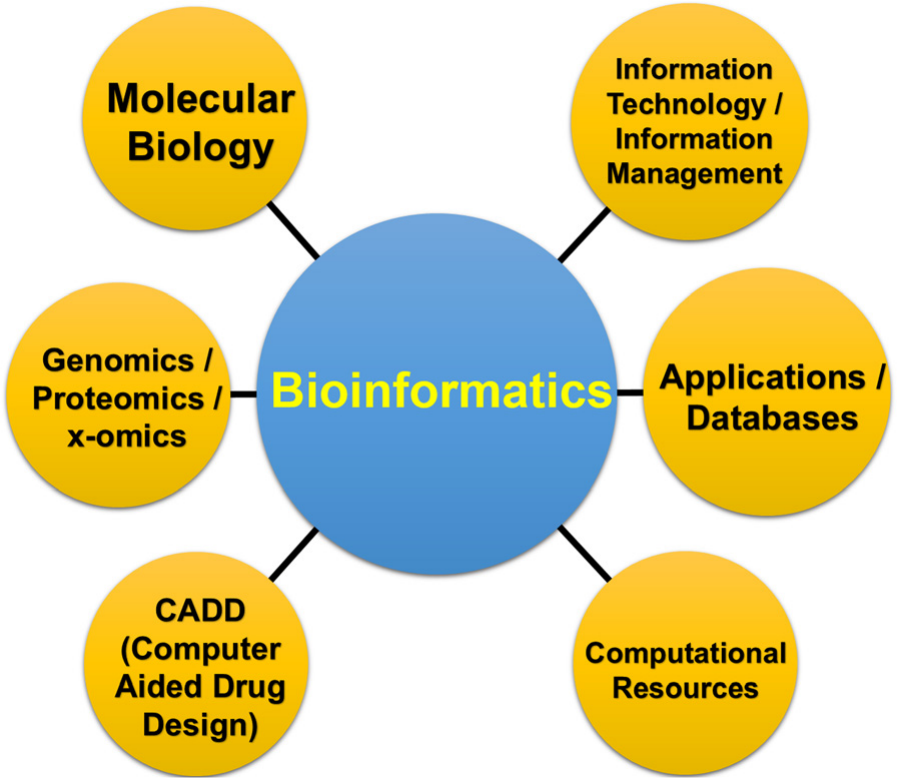
Components of bioinformatics. Genomics is a branch of science studying structural and functional organization of a genome. Proteomics is a branch of science focusing on proteins and their functions and interactions in living organisms. Metabolomics is a branch of science studying the particulars of metabolic pathways. Bioinformatics develops algorithms for the prognostication of the spatial structures of proteins and other biomolecules as well as evaluates the character of control of complex biological systems. Thanks to these informational technologies, it has become possible to model pharmaceuticals, drastically reducing the time for creation of pharmacological structures of principally new generations

Therefore, one of the important aspects of pharmaceutical design is selection of the proper PTT through which it is possible to specifically regulate some biochemical processes or complete metabolic scenarios without affecting other pathways, if possible. A considerable role in the process of development of PTT and pharmaceuticals as a whole is played by *bioinformatics*—integration of methods of applied mathematics and statistics enabling not only analysis of the available genomic, proteomic, and interactive data, but also prediction of the interaction of new level interactions on the basis of detected regularities (Fig. 7).

**Fig. 8:**
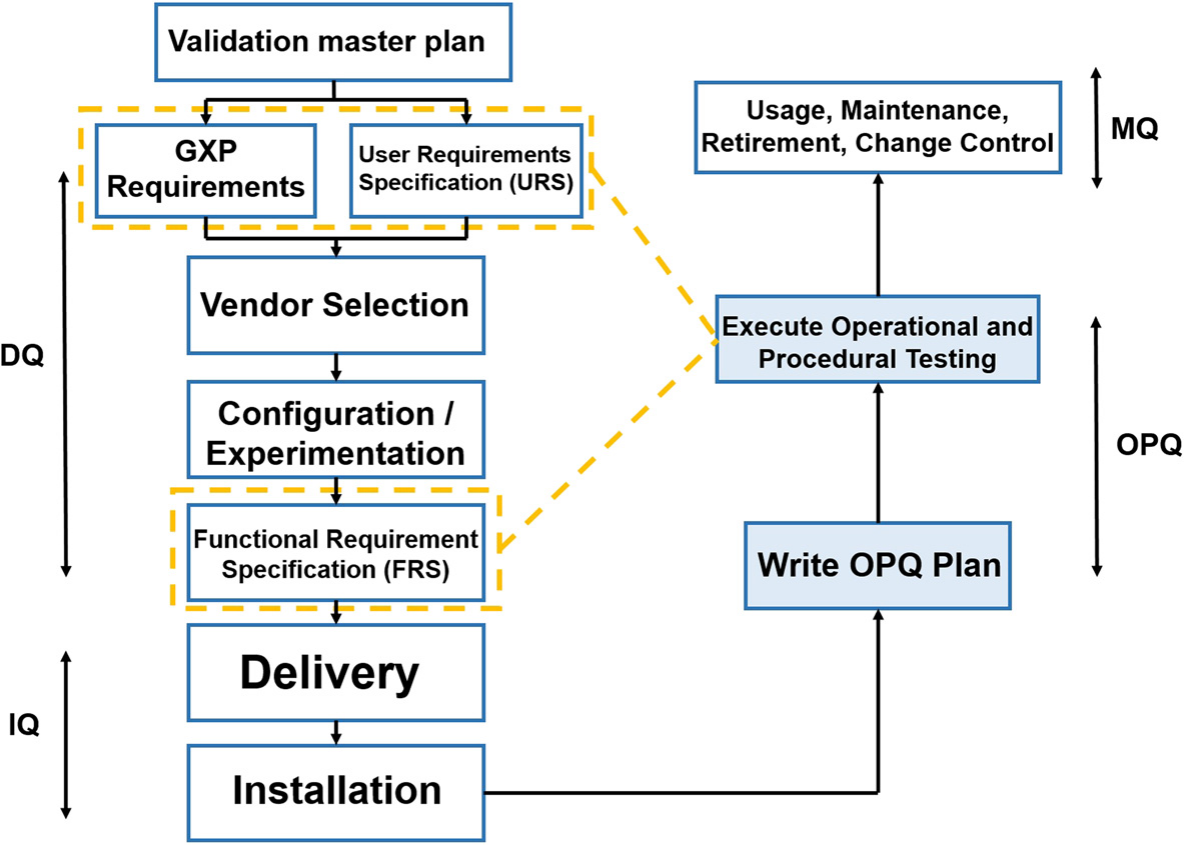
Traditional process of selecting human resources by qualification

**Fig. 9:**
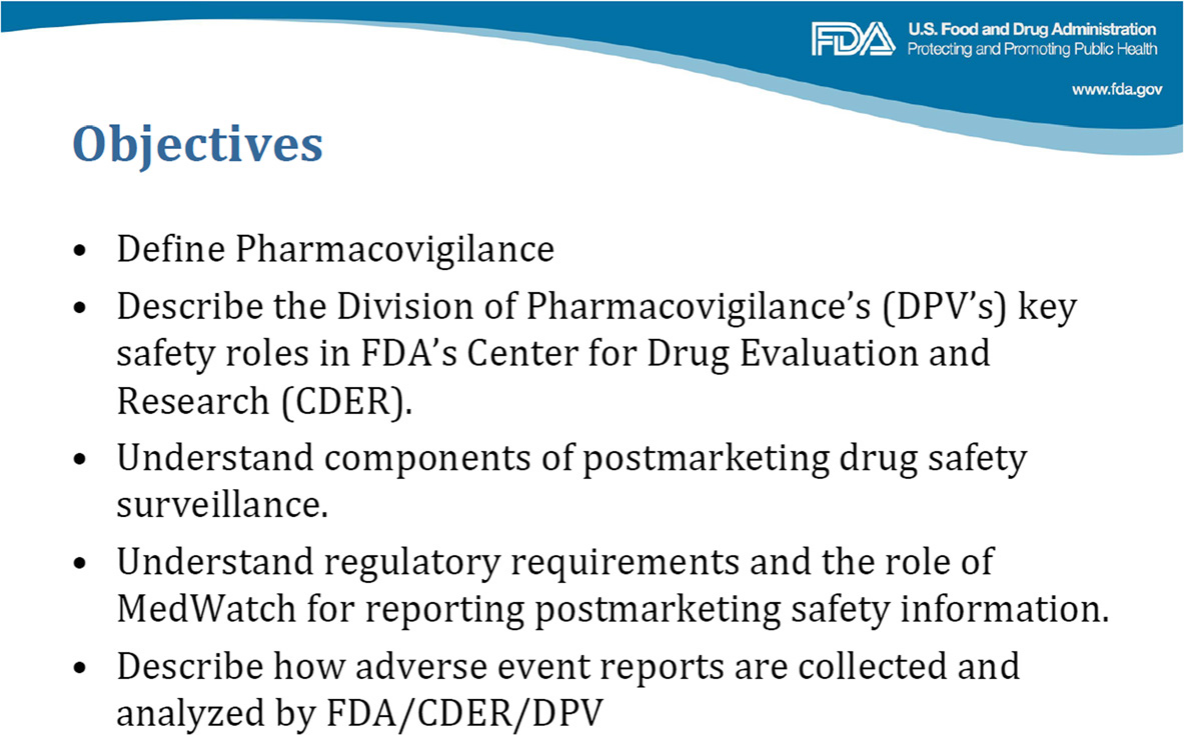
The process of qualification selection. Activities of the FDA on supervising the market release of validated pharmaceuticals

Quite recently, there has been formed a demand for new professions: *Government relations managers* (GR managers) and experts in *validation* and *pharmacovigilance* (Figs. 8 and 9), etc.

For instance, a GR manager is a specialist responsible for building productive and successful liaisons between a biopharmaceutical company and government organizations. By building up trustworthy informal relations with representatives of public organizations, GR managers promote prosperity of their enterprises and their sustainable development. A GR manager is responsible for the organization of cooperation, promotion, and protection of the interests of his/her company in the state executive bodies and public organizations. The main tasks of GR managers are to organize meetings and negotiations with representatives of state establishments, to participate in specialized exhibition shows and conferences, and to conduct seminars and presentations for representatives of state bodies. It is also the competence of the government relations manager to organize a political analysis and keep track of the legislature and political trends and relevant changes [4].

When developing and introducing new medicines and technologies into the pharmaceutical market, it is required, at each stage of a product creation, to evaluate the quality of semi-finished or finished products. In this context, a specialty of *expert in validation and pharmacovigilance* becomes highly demanded. The duties of such a specialist are to control the quality of the products manufactured including the documentary proof that the process of manufacture of the end product meets the corresponding requirements and brings the expected results, to inform medical institutions about undesirable adverse reactions of pharmaceuticals, and so on.

Development of biopharmaceutical industries is impossible without preparation of specialists on a wide interdisciplinary basis including the following specialties:

- Chemists, biologists, and bioinformatics specialists focused on applied bioengineering and developing not only drugs, but also the ideology of their creation on the basis of mathematical modeling and selection of PTT in the course of designing of therapeutic-diagnostic instruments of the future
- Pharmacologists and clinicians responsible for the conduct of preclinical and clinical research
- Technologists of a biopharmaceutical plant equipped with process lines and platforms of new generations
- Specialists in the field of certification, validation, and licensing of the end product [5]

Correspondingly, implementation of reforms in practical healthcare and naturally in the spheres of biopharmaceutical industries as their integral component requires effective measures in the policy of human resources (HR), which may allow to qualitatively change the HR potential in the biopharmaceutical industries, to adapt it to new conditions, and to execute the tasks foreseen in the reforms.

The concepts discussed above all point to the insight that for successful commercialization of a biopharmaceutical product, it is important that the specialists ensuring each stage of its development have a clear picture of the complete “path” (a translation procedure) of the medical product—from the search for a biologically active substance or creation of its virtual biostructure to the marketing of the finished product. Therefore, educational programs of medical, pharmaceutical, and technological institutions of higher education should be directed to satisfy the needs of the biopharmaceutical sector in the aforementioned specialists—a subject of great interest to the professional society.

### The role of bioengineering and biopharmaceutical industries in modern society

The achievements of modern bioengineering and biotechnology have a qualitative effect on many spheres of human activities and play an ever greater role in solving key problems of human life. Naturally, these achievements ensure controlled obtainment of useful products for various spheres of human life and are based on utilizing the functional potential of separate biomolecules, cells, whole cell systems, and living systems of various degrees of complexity.

However, a real breakthrough in the sphere of pharmaceutical design has been provided by completion of the “Human Genome Project,” leading to a totally new approach to the search and selection of potential pharmacotherapeutic targets of new generations, accessible to analytics directly in the genome text. The instrument for the analysis of such type of data massif will be bioinformatics, which has already led to a revolution indeed in biotechnology [6–8].

Clearly, progress and leadership of the USA in biotechnology and affiliated fields may be explained not only by huge investments into biopharmaceutical industries, but also by special attention to the role of professional specialists of new generations. Preparation of such specialists is one of the most important elements of the United States educational policy, the basis of which among other things is the phenomenon of intensive cooperation of university science with private pharmacological business.

Strong attention to the development of biotechnology is paid in the countries of the European Union, where the total biotechnological market has crossed the mark of 120 billion euro. Positions are also strong in South Korea, China, Japan, Great Britain, Germany, and Scandinavia.

It is precisely for this reason that the problem of preparation of specialists in bioengineering and affiliated fields is becoming particularly urgent.

### The objective necessity of educational reform in the sphere of bioengineering and biopharmaceutical industries: problems and solutions

In the world of accelerated scientific and technical progress that we are now witnessing, the today’s obsolete education model does not give *fundamental* knowledge. As discussed above, the increase in quality of training programs in universities is a priority of modern educational policy; this increase shall be based, in terms of quality assurance issues, on the role of the government in regulating, financing, and monitoring through the procedures of accreditation, licensing, and attestation [9].

Higher quality of the system of preparation of new-generation professionals implies not only the quality of the functioning system final results, but also as the quality of the process itself, which should start as early as from a pre-higher education training of future bioengineers. Special significance here should be paid to the viability of the system to select talented young specialists and promptly involve them into creative activities [10].

Then follows university education, the effectiveness of which may be evaluated with the help of the following complex criteria: the content quality of the main and post-higher educational institution educational programs and the quality of the process of realization of educational programs. At this stage, most important becomes the integration of science, education, and real production which helps integrate the resources of universities, the Russian Academy of Sciences, and business structures by way of creating various integrated structures (for instance, technology incubators, education-production centers, and others) and thus considerably increasing the effectiveness of preparation of personnel. This in turn requires the development and employment of problem-oriented technologies of biopharmaceutical education within the framework of continuous programs of specialist preparation. It becomes quite evident that higher, secondary-level and first-level education should be integrated into a single chain having the main task—to create a bioengineer of the future [11, 12].

### Issues of preparation of personnel for bioengineering and pharmaceutical design, and ways of solution

At present, one of the main problems of preparation of biotechnologists and bioengineers is the absence of modern and well-equipped bases for the production practice and, correspondingly, absence of a possibility for practical training and forming of adequate sets of professional skills with the trainees (future specialists) [13].

One of the solutions for this issue has become the introduction of federal state standards of secondary-level vocational education of the new generation. The main difference of the federal state standards of this third generation from the previous educational standards is a *modular-competence carrying* approach*.*

*Competences* is an algorithm of specialist actions which manifest themselves in practical activities and social interactions. It is exactly the module as a new structural element that holds the central place in the content of vocational education, since the requirements to the results of training are formed as a list of the types of professional activities and corresponding professional competences. A graduate of the training course should, first of all, acquire practical experience based on the complex of skills and knowledge to be mastered. Each module may be mastered independently, and their integrity permits to reach final competence in a particular professional sphere.

Complex, synchronized study of theoretical and practical aspects of each type of professional activities is performed within the framework of such modules. With regard to the abovementioned, the excessive theoretical disciplines are not so much reduced as their content is revised. That is, one envisions a kind of “sifting” of excess theory and reshuffling of the volume to emphasize the most necessary theoretical knowledge to enable mastery of the competences by arranging them in an orderly fashion and systematizing them, which, in the long run, increases the motivation of the trainees [14].

The central moment in the work to introduce the competence approach modular technology into the educational process is such forms of organization of educational activities that are based on self-dependence and responsibility for the results of work on the part of trainees themselves. The organized training process enables the student to develop self-dependence during lectures, seminars, laboratory classes, and extracurricular works, and allows independent execution of individual tasks which greatly expands one’s knowledge, develops skills, and helps develop a creative approach and ability to find one’s place in the growing flow of scientific information.

**Fig. 10:**
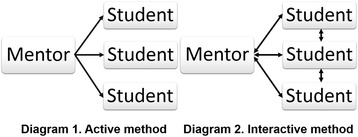
Forms of interaction of students with lecturer

One more important problem is the quality of education being received. To solve this problem, it is proposed to introduce into practice new pedagogical technologies directed at full and conscientious understanding of the material under study. One of such models is the model of interactive training of students (Fig. 10, diagrams 1 and 2) [15].

As distinguished from active methods, the interactive methods are oriented for wider interaction of students not only with the instructor, but also with one another and for the dominancy of activity of students in the process of training. Interactive material presentation should be planned beforehand, since such training requires constant control over the trainees. When the instructor addresses the students with questions or tries to involve them into discussion, he/she should always know in which direction the discussion may go and control the course of discussion. The task of the instructor is to help the group to point out a definite problem and direct the course of discussion towards clarification and solving the problem set. By using answers and considerations proposed by the students, the instructor encourages the students to work over the material and boosts up their interest in the issue under discussion in order to accentuate the correct provision. The “directed discussion” requires skill and ability on the part of the instructor to clearly set up the direction of the discussion by smoothly changing the course of discussion as well as re-phrasing the answer by giving it the right tint and stressing the right accents.

The directed discussion may be dedicated to the whole lesson, or a small series of questions-answers may be built into the speech of the instructor. An effective method to attract attention of students to important information is listing of questions at the beginning of the lecture. Besides, it is necessary to show that the instructor has noted the efforts made by the student. The main objective of the directed discussion is activation of brainwork of students and their involvement in the training process. Important difference of interactive exercises and tasks from ordinary ones is that when performing interactive exercises, students not only and not so much as make fast the already studied material, as study new material.

The interactive training expects the logics of the training process to differ from the ordinary process: not from theory to practice, but from shaping new experience to its theoretical understanding through the use of the latter. Experience and knowledge of educational process participants serve as a source of their mutual training and mutual enrichment. By sharing their knowledge and experience, the participants take upon themselves a portion of the instructor’s educational functions, thus increasing their motivation and training productivity.

An effective interactive method, very important for formation of skills for work, is demonstration. The feature of this method is the possibility to illustrate the subject under discussion for its better adoption. Thus, by using interactive methods, we set ourselves a task not only to give the students general knowledge, but also to form a definite level of expertise and skills so that in future work, particularly when using biological agents (microorganisms, plant and animal cells, and parts thereof: cell membranes, ribosomes, mitochondria, chloroplasts), future specialists in biotechnology and pharmaceutical design may feel at ease and demonstrate maturity and common sense when trying to obtain valuable products and carrying out target transformations [16].

Integration of science and education is important to increase the quality of preparation of specialists. For example, within the framework of higher educational institution structural integration, there may be integrated university departments and research institutes with similar interests to create scientific and educational complexes having a common academic council and administration system. By doing this, university lecturers do scientific work in research institutes, researchers give lessons in university departments, and the academic council coordinates all this complex work and approves educational plans of departments and research plans in research institutes.

A number of universities have already organized *education-science-innovation complexes* (ESIC) for the purpose of increasing the quality of education and strengthening the liaison with the production. The specifics of ESIC consists in that, thanks to the cooperation of scientific research and educational and production capacities, there is ensured a new quality of education, development of research, and commercialization of the results of scientific and technological joint performance.

Creation of ESIC on the basis of a structural subdivision of a higher educational institution/university (a department, laboratory of a research institute, pilot-scale production of university) without establishment or attraction of a legal entity permits to avoid problems associated with the interaction of two and more legal entities, especially when they have different forms of incorporation. In this case, it becomes easier to solve questions associated with intellectual property and organize joint educational, scientific, and innovation processes. Setting up of ESIC on the basis of a university and a large industrial enterprise permits developing long-term cooperation and preparation of duly trained personnel for the needs of the biopharmaceutical industry.

A problem of great concern is how to prepare graduates of secondary-level schools to education in universities—our potential students. Today, we are forced to admit the discrepancy between the skills of a secondary-level school graduate and the skills necessary for the fast and successful adaptation of a student to study in a university. To overcome this, we need to think about organizing a close contact in the “secondary school-university” link. In this regard, a sufficiently attractive idea is to “incorporate” the last stage of secondary-level education into the structure of higher education. We need to revive old traditions and offer universities the resources and possibilities to organize a pre-university training in our physico-technical, physico-mathematical, and ecological-biological system and other schools operating on the basis of universities. This will help to attract academic staff to teach at such schools, make use of higher school teaching technologies, and reduce the time for adaptation of students.

In addition to the creation of a “secondary school-university” cluster with the aim to increase the quality of education, a particular place in preparation of bioengineers is occupied by the “secondary school-university/college-production” cluster. The cluster pattern of this system will help prepare a competent employee for the regional labor market and achieve maximum result in a minimum time limit. Exactly, the cluster approach predetermines a mutually beneficial relationship, uninterruptedness, and cooperation. It is evident that active joint functioning of educational establishments with production facilities will create favorable conditions for increasing their investment attractiveness and competitiveness of graduates at the regional labor markets [17].

The demonstrated “good practices” uniting educational and innovation activities in universities show that with the established ties with revived or newly organized private enterprises, the scientific and educational cooperation starts its active growth. In short, the state, market and production, universities, and research institutes should promote expansion of the biopharmaceutical sector of economics, contribute to effective national industrial policy, and stimulate innovations of small businesses, thus facilitating the progress of society as a whole.

### PPPM as an innovation model of advanced healthcare and sector of biopharmaceutical industry and pharmaceutical design

The purpose of employment of the knowledge, skills, and resources of biopharmaceutical science and biopharmaceutical industry is to predict and prevent diseases, increase the life expectancy, and strengthen and preserve human health through organized efforts of society.

The essence of the new model of advanced healthcare—PPPM*—*is in guiding the human body reserves, and its main purpose is not healing the diseases, but detecting concealed anomalies in organisms and taking targeted measures directed at the liquidation of such anomalies and preventing diseases. Naturally, such type of global change could be obtained only with active introduction of fundamental science achievements into life, making it possible to get inside the affected biostructures with conditions to visualize the damaged sites (including those concealed from the doctor’s eyes) with further liquidation thereof at the preclinical stage and thus preventing the beginning of disease.

Today, we already observe growth in the share of predictive and preventive medications in the overall spectrum of developed and manufactured drugs. This requires organizing, in the medical and chemico-pharmaceutical higher educational institutions, special educational programs incorporating technological and other aspects of modern biopharmaceutics including the tasks of PPPM-affiliated branches of the biopharmaceutical industry [18].

In this regard, the reform of pedagogical process and creation in the structure of medical universities of relevant chairs and later PPPM departments will reflect the systematic approach to form innovation infrastructures with the purpose to modernize the present-day profile of the biopharma industry. The requirements to the specialists of the future, i.e., specialists in the field of pharmaceutical design, traditional medicine, and bioinformatics, will also change.

Correspondingly, at this stage, the training programs should include the tasks of training the students and planting skills in the following fields:

- Understanding of the key molecular mechanisms of disease development and designing models of pathophysiologic mechanisms of the latter with preliminary selection of potential pPTT
- Identification of basic structural-functional shifts in the physiological architectonics of cell biomolecules causing generation of cell pathology, pathology of intercellular interactions, and, as a result, the overall pattern of clinical symptomatics
- Screening of biomarkers necessary for use in predictive diagnostics, prognostication, and monitoring of diseases at the preclinical and clinical stages of disease
- Understanding of the principles of modern diagnostics, and analysis and interpretation of laboratory data permitting to perform identification of key cellular shifts when various pathologies are formed
- Use of molecular targets with the aim of prophylactics and prevention of disease at the clinical stage or typical pathological process at the preclinical stage

A graduate of specialized courses should naturally know the following:

- Theoretical and methodological basis of fundamental medicine corresponding to the contemporary level of world knowledge in the field of systems biology and fundamental biomedicine
- Principles of designing of a model of pathological process with identification of biomarkers and selection of targets necessary for effective control of pathological process
- Pool of modern technologies used to improve the effectiveness of the analytical cascade including the use of interdisciplinary approaches and modernization of the integrated infrastructure of the biopharma industry

The PPPM specialists should be experts not only in interdisciplinary (legal, organizational, and statistical) fields of action, but also in the fundamental spheres that offer, on the one hand, a clue to the principally new pharmaceuticals and diagnostic technologies, and, on the other hand, personalization of diagnostic reports, prevention, and prophylactics. Such researchers-experts should possess technologies of planning and standardization, and their preparation requires restructuring of programs for pre-university (school), university, post-graduate, and basic medical education with the formation of principally new interdisciplinary programs which will be oriented for training and retraining of specialists in the PPPM-affiliated directions.

Correspondingly, preparation of specialists capable of building the interdisciplinary healthcare system of the future should be built on the novel principles taking into account the following:

- Common architectonics of pre-university, university, and post-graduate education
- Features of introducing a *secondary school-university* pair into international educational environment
- The role of additional or further education and bioinformational technologies as the basis of such environment
- Principles of design in the structure of additional education and design of business, research, and engineering
- A rigid necessity of a multiple-level testing and dialog in the *learner-teacher* pair with due account of personality characteristics of the two when forming vocational self-sufficiency and professional potential of a specialist as a personality of the future
- The value of innovation risks of the educational process and possibility to monitor such risks in case of emergency situations

This educational model should contain the following:

- Educational-methodological kernel
- Key platforms of basic knowledge and qualities
- A system of group and individual vectors demonstrating priority directions and substantive potentials of intensiveness and quality of knowledge development

Thus, for instance, the first (pre-university) level of education familiarizes school children with the modern model of PPPM (without deep study of any separate aspects).

The second (university) level implies deep studying of fundamental (theoretical) and applied (translational) aspects of PPPM among the students.

The core of the third (post-graduation) level is dedicated to interdisciplinary aspects of PPPM affiliated to resident doctors and post-graduate students.

In this respect, the starting point of training in the field of high technologies of school children is of particular importance (mainly senior school children), this training being organized on the basis of leading academic and industry research institutes. The procedure of forming a specialist and upbringing his/her creative personality should not only be solved at the stage of post-university adaptation in real work, but also start at the stage of training sessions in the pre-university (at school) phase with simultaneous conduction of specialized workshops. And, since the school program is of great importance to the future education and vocational careers of our specialists, it is necessary to unite efforts of teachers, administrators of universities, and political leaders for the development of educational programs on modern biopharmaceuticals in the secondary-level schools and colleges [19].

### A team of young scientists—our hope in the development of domestic biopharma industry

**Fig. 11:**
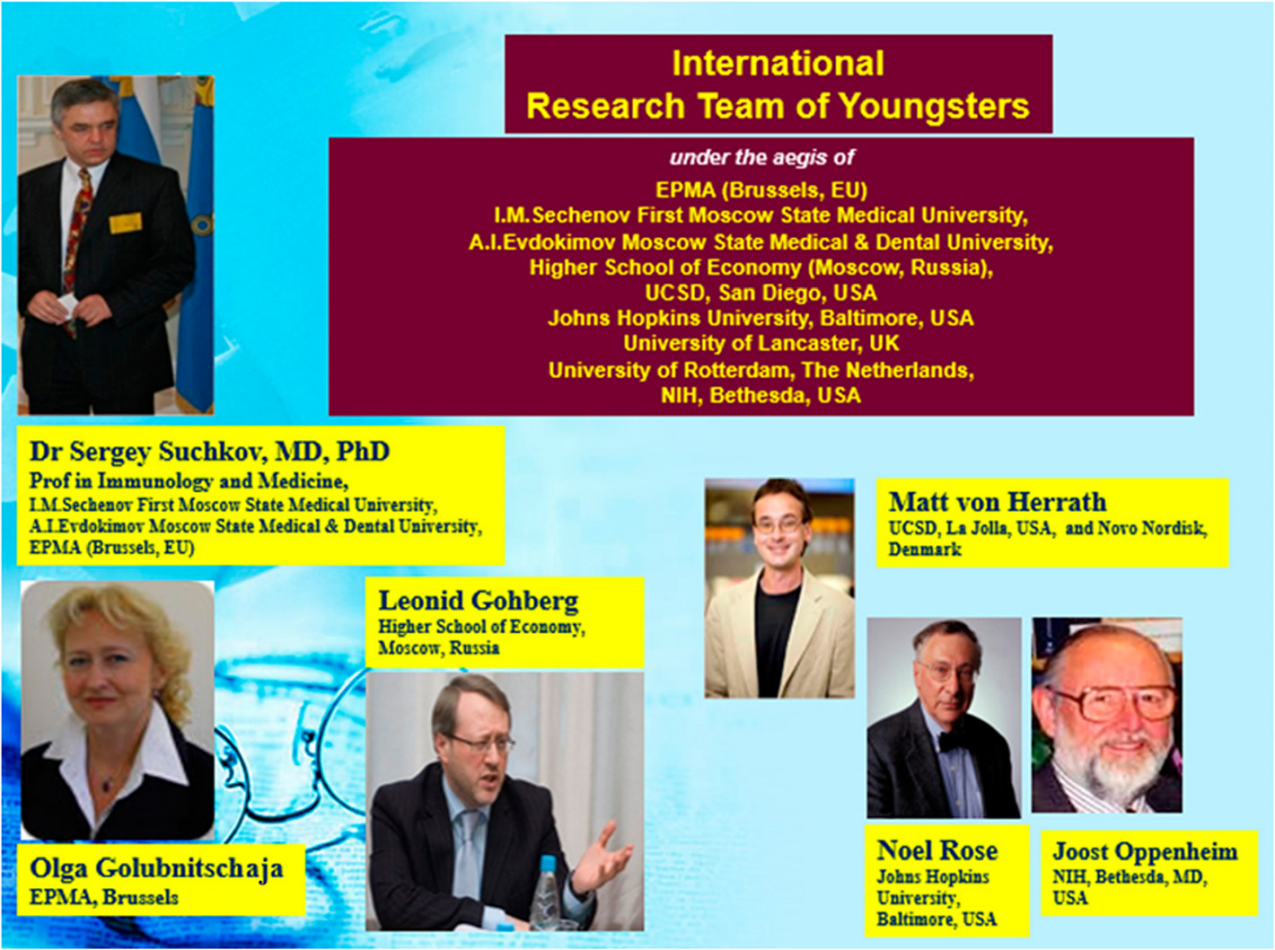
Creators-founders of the international young scientists team

**Fig. 12:**
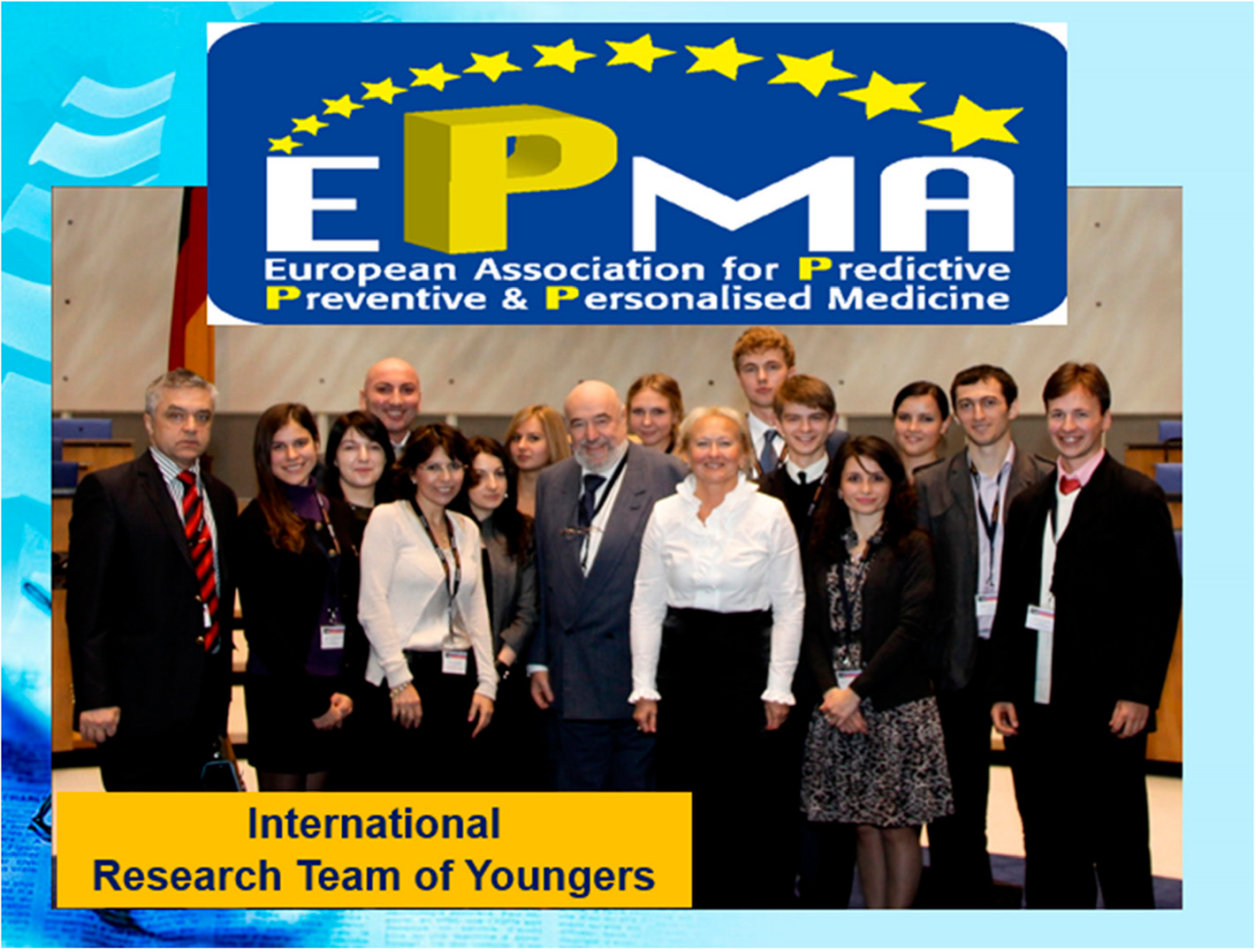
Members of the international young scientists team at the First International Congress on PPPM, Bonn, Germany, 2011

**Fig. 13:**
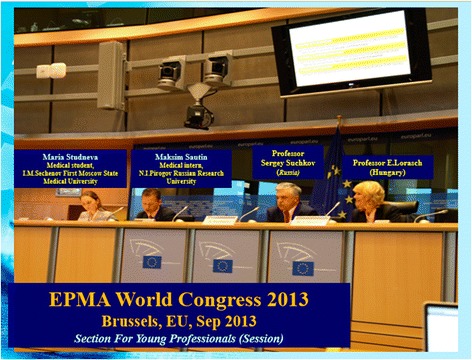
Members of international young scientists team at the Second International Congress on PPPM during discussion of training program results, Brussels, European Parliament, 2013

Taking into consideration the current trends and personal experience, we have made first steps for closer liaison of the secondary school-university pair and restructuring of specialized (medical-biological) classes to turn school children towards direct participation in the modernization of the biopharmaceutical industry system. Guided by the abovementioned facts, a quite non-standard approach has been the organization of a young scientist team which has acquired an official status with the international research group in the European Association for Predictive, Preventive, and Personalised Medicine (EPMA). The characteristic feature of this group was that in addition to the resident doctors and students of Moscow universities, the group comprised the first school children of Education Center No. 204 named after А.M. Gorky (a Federal State-Funded Educational Institution) and students of non-medical universities having interest in allied profession (Figs. 11, 12, and 13).

Used as an educational-methodical kernel is a three-level basic education system (pre-university, university, and post-university education). The genome and post-genome technologies have occupied a special place among the basic platforms as well as methods of cellular and tissue-specific bioengineering. Group and individual vectors as part of the basic inventory are represented by the branch segments including pharmaceutical design, translational medicine, bioinformatics, and others—i.e., vectors incorporating all modern technological platforms.

Attention should be paid to a number of essential moments which appeared as a result of the first experiments. First*,* not only students of universities, but also individual school children have started their research work by selecting leading academic research institutes, and as the subject of research, they have chosen most recent technologies relating to genome and post-genome topics requiring from the specialists-bioengineers many a year of continuous training for forming corresponding competences.

Second, one of the most pressing and controversial problems in the system of innovation specialist preparation is the problem of adaptation of university graduates in the real production environment, i.e., at innovation biopharmaceutical enterprises connected with research centers that generate innovation products. Here, for senior school age, the leading type of activities is the design and research activity as a means of his/her professional self-determination; hereby, the “object of design” is the schoolchild who designs (“forms”) his/her abilities necessary to master the chosen profession. Actually, as for schoolchildren, this is the research work which gives rise to love of sciences and the creative profession of a bioengineer.

In work with gifted children, the innovation technologies occupy a special place, since the purpose of such work is the development of intellectual and creative potential of the child personality by developing the research abilities of the latter. The results of mutual work and with gifted children and the relevant educational process has been taken by us as the basis for the evaluation of the results of the design work of the student-schoolchild pair or the post-graduate student-student-schoolchild triplet.

## Conclusions

Our model for accelerated development of continuous vocational education in the sphere of biopharmaceutics and biopharmaceutical industries is based on the combinatorial approaches (competence, module, personality-activity, program-design, and problem-oriented) to the elucidation of innovative processes of modernization of the existing system. Correspondingly, the unit to build up the content of educational programs and sites is the task of pedagogics oriented for the innovation context in education development, and it allows each hearer to organically combine individual and group work with the aim to enrich oneself with the experience of the colleagues and also to use own professional experience.

The use of the accelerated model for development of continuous vocational education has required a new type of organization of the educational process. The place of formalized methods and means of education focused on the transfer and learning of information is now occupied by innovation and interactive, problem- and practice-oriented, and research-and-design methods. All that ensures solving of pedagogical problems on the basis of constructive dialog, exchange of opinions, role and positional interaction, practical solution of educational tasks, and use of information technologies.
